# Resections of the Hip-joint in War Wounds

**Published:** 1918-12

**Authors:** 


**Resections of the Hip-joint in War Wounds.** By E. Potherat. *Bull. de la Société de Chirurgie de Paris*, June 25, 1918.

The available method of operating the hip-joint is quite different whether the shaft, neck and head of the femur, although injured, continue to be firmly united and solid, or whether the shaft is separated from the head.

In the first case, the operative mode is the following one : the capsula being split along its posterior vertical axis, the trochanterian insertions are carefully loosened; the head is then separated easily from the acetabulum, and the hip disjointed by means of forced movements impelled on the femur. Next, the injured head and necessary length of the bone shaft can be sawed away.

In case only the head of the femur is broken, no great difficulty arises, the fragments of the bone being taken off one by one by means of forceps, and then the neck being easily disjointed and resected.

But supposing the neck itself should be completely broken off and no longer solidly united to the shaft, this cannot act any more as a lever to disjoint the hip. Resection " in situ " must be performed, then, by slow removal of each broken piece of bone. This removal takes place in the bottom of a somewhat deep hole, between hardened tissues, presenting no elasticity at all, and therefore very difficult to pull aside in order to widen the aperture effected between the muscles. This always proves a hard task, even if undertaken soon after the wound occurs; but it proves much harder still if a long time has elapsed between the injury and the operation.

Such was the case for a patient operated upon by Dr. Potherat. This man was wounded in May, 1917, and a bullet fractured his left thigh-bone. X-ray showed a fracture of the neck extending to the large trochanter; Dakin's solution was injected by means of a drain and traction applied to the thigh. Suppuration took place, proving the wound to be infected. Resection was being resorted to when the patient happened to be removed into another hospital. There the Carrel-Dakin treatment was carried on for five months. Then X-ray seemed to show some fair consolidation of the bone, this, however, being a false diagnosis as was recognized later on. For five months more the wound was left suppurating abundantly, the patient being feverish and his condition very poor. He could not rise from his bed and the hip-joint was quite stiff.

Dr. Potherat happened to examine that patient in March, 1918, 10 months after the injury had occured. He diagnosed immediately a persisting suppurating focus into the fracture, and resection was decided.

No operation, states the author, ever proved so difficult; the large trochanter was enclosed, together with the neck and head of the femur, into a lump of lardaceous tissues, four fingers thick in some places, impossible to pull aside to lay the bones bare. Finally that tissue had to be partly cut off, sufficiently to allow the attack of the bones by means of a gouge, and the progressive removal of the fragments.

The head, neck, and a large trochanter were removed in that way. The operation lasted for an hour and a half, ether being used for anesthesia.

The wound was left wide open and injected with magnesium chloride. The repairing process proved very active, and 6 weeks later the wound was closed.

